# MR thermometry for focused ultrasound monitoring utilizing model predictive filtering and ultrasound beam modeling

**DOI:** 10.1186/s40349-016-0067-6

**Published:** 2016-09-22

**Authors:** Henrik Odéen, Scott Almquist, Joshua de Bever, Douglas A. Christensen, Dennis L. Parker

**Affiliations:** 1 Utah Center for Advanced Imaging Research, Department of Radiology, University of Utah, Salt Lake City, UT USA; 2 School of Computing, University of Utah, Salt Lake City, UT USA; 3 Department of Bioengineering, University of Utah, Salt Lake City, UT USA; 4 Department of Electrical and Computer Engineering, University of Utah, Salt Lake City, UT USA

**Keywords:** MR thermometry, Ultrasound modeling, Model predictive filtering, Hybrid angular spectrum, Proton-resonance frequency shift

## Abstract

**Background:**

A major challenge in using magnetic resonance temperature imaging (MRTI) to monitor focused ultrasound (FUS) applications is achieving high spatio-temporal resolution over a large field of view (FOV). This is important to accurately monitor all ultrasound (US) power depositions. Magnetic resonance (MR) subsampling in conjunction with thermal model-based reconstruction of the MRTI utilizing Pennes bioheat transfer equation (PBTE) is one promising approach. The thermal properties used in the thermal model are often estimated from a pre-treatment, low-power sonication.

**Methods:**

In this proof-of-concept study we investigate the use of US simulations computed using the hybrid angular spectrum (HAS) method to estimate the US power deposition density *Q*, thereby avoiding the pre-treatment sonication and any potential tissue damage. MRTI reconstructions are performed using a thermal model-based reconstruction method called model predictive filtering (MPF). Experiments are performed in a homogeneous gelatin phantom and in a gelatin phantom with embedded plastic skull. MPF reconstructions are compared to separate sonications imaged with fully sampled data over a smaller FOV. Temperature root-mean-square errors (RMSE) and focal spot positions and shapes are evaluated.

**Results:**

HAS simulations accurately predict the location of the focal spot (to within 1 mm) in both phantoms. Accurate temperature maps (RMSE below 1 °C), where the location of the focal spot agrees well with fully sampled “truth” (to within 1 mm), are also achieved in both phantoms.

**Conclusions:**

HAS simulations can be used to accurately predict the focal spot location in homogeneous media and when focusing through an aberrating plastic skull. The HAS simulated power deposition (*Q*) patterns can be used in the MPF thermal model-based reconstruction to obtain accurate temperature maps with high spatio-temporal resolution over large FOVs.

## Background

Focused ultrasound (FUS) treatments are currently being performed under both ultrasound (US) and magnetic resonance imaging (MRI) guidance. One of the main advantages of MRI guidance is the ability to accurately measure and monitor the temperature evolution in real-time using MR temperature imaging (MRTI) [1, 2]. MRTI enables evaluation of the treatment target site in terms of measuring temperature rise and thermal dose, as well as safety monitoring of the surrounding healthy tissues in the US near and far field. The current gold standard in MRTI is the proton-resonance frequency shift (PRFS) method, which relies on the temperature-dependent shielding of the hydrogen nucleus from the surrounding electron cloud. The shielding effect increases with temperature as hydrogen bonds between water molecules stretch, bend, and break, resulting in a lower resonance frequency with increased temperature [3–6]. The main advantages of the PRFS method are its relatively high sensitivity, that it can be monitored with readily available MRI pulse sequences, and that it is to a large degree tissue type independent for most aqueous-based tissues [7]. One drawback of the PRFS method is that it does not work in lipid-based tissues since these lack the necessary hydrogen bondings.

One of the main challenges with MRTI is to achieve accurate temperature imaging with sufficient temporal and spatial resolution [8] over a large enough field of view (FOV) to monitor all surrounding and intervening tissues for unwanted heating. This is especially challenging in FUS applications since the US energy can be focused deep into the body. Attempts to increase the acquisition speed and/or volume coverage for MRTI have included using fast pulse sequences such as echo planar imaging (EPI), where multiple k-space lines are acquired after each radiofrequency (RF) excitation. Both single- and multi-slice 2D as well as fully 3D versions, with both single-shot and segmented phase encodings, have been investigated [9–15]. Other fast pulse sequences that have been investigated include balanced steady-state free precession (bSSFP)-type sequences which have a relatively short repetition time (TR) [16, 17], as well as utilizing signal-to-noise ratio (SNR)-efficient non-Cartesian approaches [18–21]. Speedups have also been achieved by combining subsampled k-space data acquisition with subsampled data reconstruction methods such as parallel imaging [22–26], compressed sensing [27–29], and temporally constrained reconstruction approaches [30, 31]. Filtering approaches, such as Kalman filters, and fitting and modeling methods, such as thermal modeling utilizing the Pennes bioheat transfer equation (PBTE), have been used to reconstruct temperature images based upon limited k-space data samples or as a mean of noise reduction in fully sampled data [19, 32–35].

Model predictive filtering (MPF) is a previously published thermal model-based method for reconstructing subsampled MRTI data for FUS applications. MPF uses thermal modeling through the PBTE to forward predict temperature change. The temperature change is converted to a change in the MR phase image and then used to fill in missing data in k-space from the subsampled acquisition. MPF can achieve high spatio-temporal resolution over large FOVs, but the thermal modeling requires estimates of both tissue thermal and acoustic properties. These properties, such as thermal conductivity, *k* (in W/m/°C), and deposited US power density, *Q* (in W/m^3^), can be estimated from a pre-treatment low-temperature rise sonication [36, 37]. While these test sonications currently serve purposes to localize the focal spot and ensure good US coupling, they also result in additional, potentially harmful tissue heating [38–40] and may also prolong the total treatment time.

In this study, we use US modeling with the previously published hybrid angular spectrum (HAS) method [41–46] to obtain the US power deposition (*Q*) required for MPF [32, 47, 48]. This avoids the potentially harmful pre-treatment heating and can result in shorter total treatment time since the treatment can be planned and *Q* can be calculated beforehand. In this proof-of-concept study, experiments are performed in a homogeneous gelatin phantom and in a gelatin phantom with an embedded plastic skull. In the skull phantom, HAS-based US simulations are also used for phase aberration correction using a previously described method [49].

## Methods

### MR imaging and image reconstruction

All MR imaging was performed on a 3T MRI scanner (TIM Trio, Siemens Medical Solutions, Erlangen, Germany) with imaging parameters summarized in Table 1. A 3D gradient recalled echo (GRE) pulse sequence with segmented EPI readout was used for all MRTI. k-space was subsampled with a Cartesian variable-density equally spaced sampling scheme [50], where the sampling in the kz-slice encode direction was varied to achieve denser sampling over the k-space center while maintaining a constant echo-spacing down the echo train. The mask used to segment out the skull for the phase aberration correction (see below) was based on a higher resolution standard 3D GRE scan (i.e., no EPI read out). All MR data were zero-filled interpolated to 0.5-mm isotropic voxel spacing to minimize partial volume effects [51].

**Table 1:** MR scan parameters

	Scan	TR/TE (ms)	FOV (mm)	Voxel size (mm)	BW (Hz/px)	ETL	ES (ms)	FA (°)	*t*_acq_ (s)	*R*
Homogeneous phantom	“Truth”	22/11	147 × 96 × 50	1.2 × 1.2 × 2.5	752	7	1.62	15	4.8	1
	Subsampled	22/11	147 × 110 × 135	1.2 × 1.2 × 2.5	752	7	1.62	15	2.4	7
Skull phantom	“Truth”	22/12	288 × 189 × 45	2.3 × 2.3 × 2.5	752	7	1.62	15	4.8	1
	Subsampled	22/12	288 × 221 × 135	2.3 × 2.3 × 2.5	752	7	1.62	15	2.4	7
	High res. mask	10/5	288 × 288 × 90	1.2 × 1.2 × 1.3	752	1	N/A	10	104	1

*TR* repetition time, *TE* echo time, *FOV* field of view, *BW* bandwidth (in readout direction), *ETL* echo train length, *ES* echo spacing, *FA* flip angle, *t*_acq_ acquisition time, *R* k-space reduction/subsampling factor (*R* = 1 means fully sampled)

**Fig. 1:**
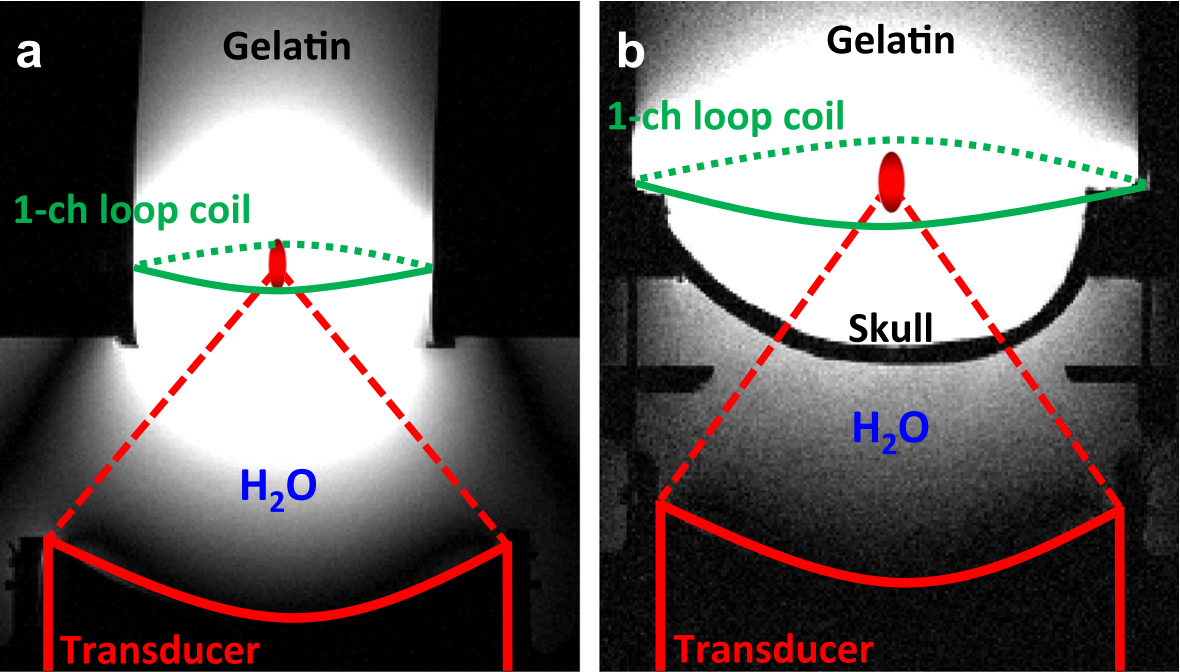
Experimental setup. Scan setup of **a** homogeneous gelatin phantom and **b** gelatin phantom with embedded plastic skull. The ultrasound transducer was positioned below the phantoms and coupled with a bath of de-ionized and de-gassed water

Two in-house-built RF receive-only single-channel loop coils (schematically shown in green in Fig. 1) with different diameters (12 cm for the homogeneous phantom and 18 cm for the skull phantom, respectively) were used for signal detection.

The MRTI data was reconstructed with both the MPF method and using a previously published compressed sensing-like temporally constrained reconstruction (TCR) algorithm for comparison [15, 30, 52]. Full descriptions of the MPF and TCR algorithms are given elsewhere [30, 32], and only a brief description as background is given here. The MPF algorithm utilizes the PBTE [53] given by the following:

1 $$ \rho {C}_t\frac{\partial T}{\partial t}=k{\nabla}^2T-W{C}_b\left(T-{T}_{\mathrm{blood}}\right)+Q, $$

where *ρ* = tissue density (kg/m^3^); *C*_*t*_, *C*_*b*_ = specific heat of tissue, blood (J/kg/°C); *T*, *T*_blood_ = tissue, arterial blood temperature (°C); *k* = thermal conductivity (W/m/°C); *W* = Pennes perfusion parameter (kg/m^3^/s); *Q* = US power deposition density (W/m^3^). This equation is used to forward predict the temperature change from one dynamic MR time frame, (*n*), to the next, (*n + 1*), in 3D. The 3D temperature change map is converted to a corresponding phase change map using the PRFS equation (see Eq. 2) and is then combined with the magnitude image from the previous dynamic time frame (*n*) to create a complex image. The complex image is Fourier transformed into k-space and used to fill in the parts of k-space that were not sampled during time frame (*n + 1*). This updated k-space is finally inverse Fourier transformed back to image space, where updated temperature maps can be calculated using the PRFS equation.

The TCR algorithm iteratively adjusts predicted images to minimize a cost function consisting of a fidelity term and a constraint term. The fidelity term ensures that the solution is faithful to the acquired subsampled k-space data, and the constraint term applies an appropriate temporal smoothness constraint, in this work utilizing the L1-norm.

All MR temperature maps were calculated with the PRFS method, where the phase difference between two images, *Δφ*, is scaled to yield the temperature difference, *ΔT*, according to:

2 $$ \varDelta T=\frac{\varDelta \varphi }{\gamma \alpha {B}_0\mathrm{T}\mathrm{E}}, $$

where *γ* is the gyromagnetic ratio (Hz/T), *α* is a thermal coefficient (here assumed to be −0.01 ppm/°C), *B*_0_ is the main magnetic field strength (T), and *TE* is the echo time (s). Since all FUS sonications were relatively short (<30 s, see below), no field drift correction was applied. All image reconstruction and post-processing was performed using MATLAB (R2015a, The MathWorks Inc., Natick, MA).

The subsampled MRTI data for both phantom experiments were reconstructed in three ways: (1) with MPF using *Q* from HAS US simulations (see below) and previously published phantom properties (*ρ, C*_*t*_*, k)* [54]; (2) with MPF using *Q* and *k* determined from fitting a pre-treatment, low-power sonication (i.e., low-temperature rise) using a previously published analytical solution of the heating [36, 37, 55] together with the previously published phantom properties (*ρ, C*_*t*_*)* [54]; and (3) using the TCR algorithm. The three methods are referred to as *MPF HAS*, *MPF LP analytical fit*, and *TCR*, respectively, in the remainder of this paper. The three reconstruction methods for the subsampled data were compared to an estimate of the “true” temperature rise obtained using identical US sonications monitored with fully sampled k-space data over a smaller FOV (Table 1). The short-duration, low-temperature rise test sonications used in MPF LP analytical fit to analytically obtain *Q* and *k* were imaged with the same MR parameters and fully sampled data as the “truth” sonications (Table 1).

### Phantoms

Both phantoms were fabricated from 250-bloom gelatin [54] [speed of sound = 1553 ± 10 m/s (mean ± standard deviation), US attenuation = 0.06 ± 0.01 Np/(cm*MHz), density *ρ* = 1057 ± 44 kg/m^3^, specific heat capacity *C*_*t*_ = 3635 ± 88 J/(kg*°C), thermal conductivity *k* = 0.55 ± 0.01 W/(m*°C)] using previously published methods [54]. The mean values listed were used for the US simulations (see below). The plastic skull used in this study was commercially purchased (model A20, 3B Scientific, Tucker, GA, USA) and composed of homogeneous PVC plastic with varying thickness. Tabular acoustic properties for the PVC material were used in the HAS simulations [speed of sound = 2376 m/s, US attenuation = 1.50 Np/(cm*MHz), density *ρ* = 1200 kg/m^3^ [56, 57]].

### FUS sonications

FUS sonications were performed using an MR-compatible phased-array ultrasound transducer (256 elements, 1-MHz frequency, 13-cm radius of curvature, focal spot size full width at half maximum 2 × 2 × 8 mm, Imasonic, Voray-sur-l’Ognon, France), accompanying hardware and software for mechanical positioning and electronic beam steering (Image Guided Therapy, Pessac, France), and in-house developed hardware and software (code written in MATLAB) for applying the phase aberration correction. The transducer was coupled to the phantoms with a bath of de-ionized and de-gassed water as indicated in Fig. 1.

In the homogeneous phantom, three continuous-wave sonications at 40 W for 28.7 s were performed for both the subsampled and the fully sampled truth cases, for a total of six sonications. For experimental *Q* and *k* identification using the LP analytical fit method, a low-temperature rise continuous-wave sonication at 14 W for 28.7 s was performed. Similar experiments were performed in the skull phantom, at 125 W for 23.9 s (three continuous-wave sonications each for fully sampled truth and subsampled k-space, for a total of six sonications) and at 100 W for 14.3 s (LP analytical fit).

### US simulation and phase aberration correction

The US simulations were performed with the HAS method [41–43]. HAS is a full-wave simulation method that takes into account US refraction, absorption, and first-order reflection. Inhomogeneous tissues are modeled by segmenting the anatomy into a 3D Cartesian grid and assigning unique speed-of-sound, absorption coefficient, and density values for the tissue type within each voxel. The hybrid angular spectrum approach leap-frogs between the space and spatial-frequency domains, utilizing the space domain to account for individual voxel attenuation and speed-of-sound properties and the spatial-frequency domain to linearly propagate the US wave from plane-to-plane throughout the model. One of the main advantages of HAS is the short computation time, which can be up to two orders of magnitude shorter than with, e.g., finite-difference time-domain (FDTD) simulations [49].

Due to differences in the speed of sound in the skull and gelatin, the US waves from the different elements in the phased-array transducer will arrive at the US focus out of phase with each other. To correct these phase aberrations, HAS calculates the individual pressure pattern of each of the 256 elements in the phased-array transducer at the intended focal spot location, with an initial assumption of all elements having zero phase and the same amplitude [49]. Maximum constructive interference at the focal spot is then achieved by compensating for the phase offsets of each element. The computation for each of the 256 transducer elements was performed in parallel on a GPU (Nvidia Tesla, Nvidia, Santa Clara, CA) for maximum computation speed, and the full phase correction simulation took approximately 5 min. Once the correct phases for all 256 elements have been found, a single HAS simulation is done to find the *Q* pattern, and this takes approximately 10 s (this can be compared to the time it takes to perform the analytical fit for MPF LP analytical fit, which is on the order of minutes [58]). Since HAS is a full-wave simulation method, US amplitude and phases are calculated in a full 3D volume, so treatment planning and phase aberration correction for multiple and arbitrary positions in the full volume can be achieved with minimal added computation time.

For the homogeneous phantom in this study, a two-compartment segmentation consisting of water and phantom material (gelatin) was performed based on 3D segmented EPI magnitude images. Based on this segmentation and the distance from the transducer to the bottom of the phantom, measured in the same images, HAS was applied to simulate the 3D *Q*-pattern inside the phantom. For the skull phantom study, the segmentation was based on a high-resolution 3D GRE scan (i.e., no EPI readout) and a three-compartment segmentation consisting of water, phantom material (gelatin), and skull was performed.

### Evaluation of accuracy and precision

The root-mean-square error (RMSE) was used to compare the accuracy of the three different reconstruction methods. The mean of the three fully sampled datasets was used as the reference truth for comparison to all other reconstruction methods (the three truths were acquired interleaved with the three subsampled datasets). RMSEs were calculated for the hottest voxel (HV) in each sonication as well as for a global error around the focal spot, defined as all voxels having a temperature rise greater than 15 % of the hottest truth voxel, and for a more local error around the focal spot, defined to be all voxels having a temperature rise greater than 85 % of the hottest truth voxel. Percentage temperature rise relative to truth was chosen since the temperature rise in the homogeneous phantom was almost twice as high as in the skull phantom, and it was deemed that this would be a more comparable measure between the two phantoms than the RMSE for all voxels above an absolute temperature limit. Since the temporal resolution of the subsampled scans was twice as high as truth (2.4 vs. 4.8 s, see Table 1), and the temperature measured with MRTI should be assigned to the time when the center of k-space is sampled (which for segmented EPI pulse sequences occurs half way through the acquisition), linear interpolation between two consecutive subsampled scans was used to estimate the temperature at the time corresponding to when the fully sampled acquisition sampled the center of k-space. The linearly interpolated values were used when calculating the RMSE. The mean and standard deviation (SD) of the RMSE for the three repeated sonications were calculated for both phantoms.

For all sonications, the focal spot center and the full width at half maximum (FWHM) of the temperature profiles were computed and the MPF and TCR values were compared to truth. All FWHM values were found by linear interpolation within the zero-filled interpolated voxels.

*Q*-patterns as obtained from the HAS simulations and the analytical fits to the LP pre-treatment sonications were also calculated and compared for the two phantoms. Maximum values and FWHM were compared between the two methods (i.e., HAS and LP analytical fit), and positions were compared to truth heatings.

## Results

**Fig. 2:**
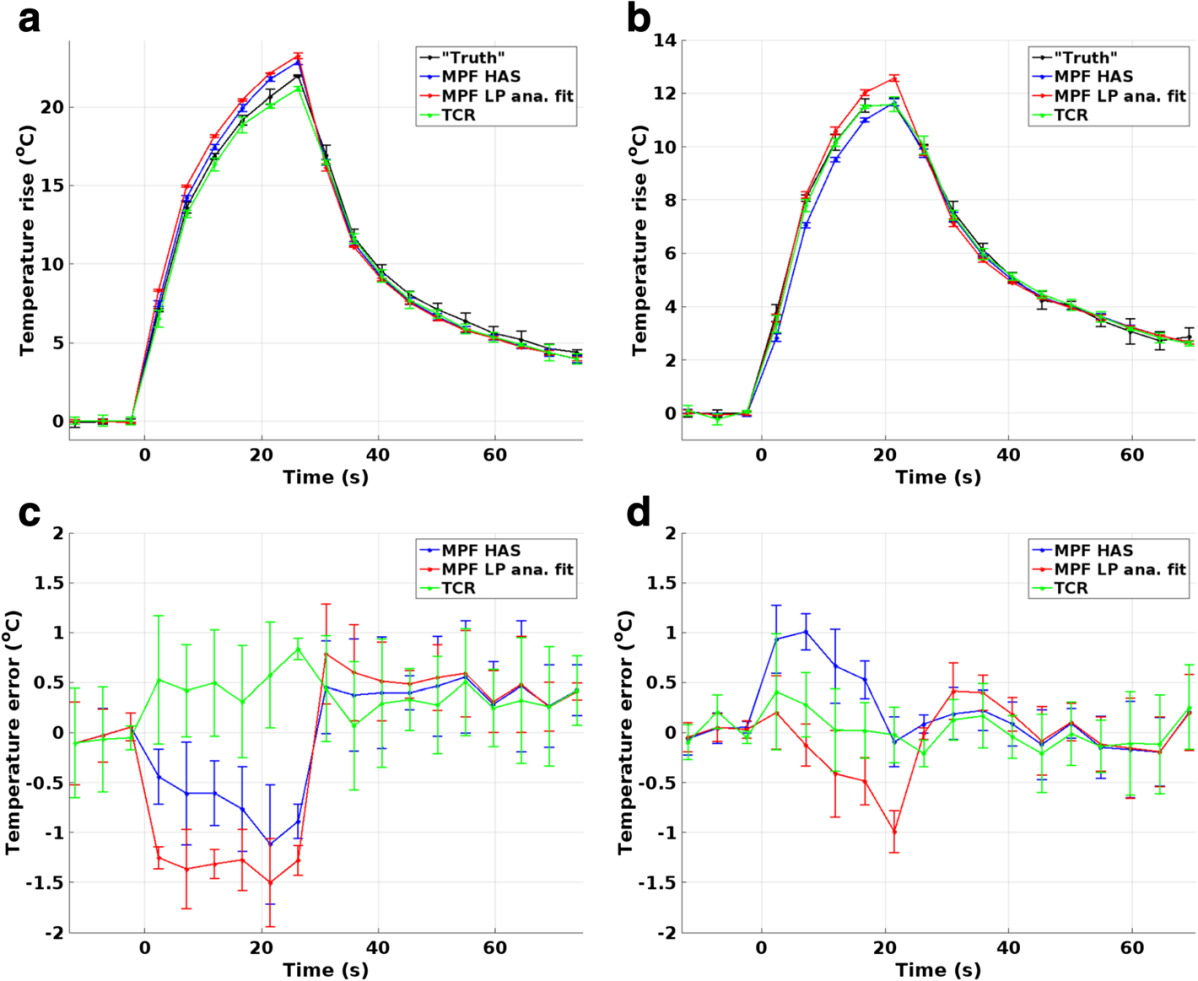
Temperature versus time plots. Mean and SD of temperature rise versus time for the hottest voxel for “truth” and the three reconstruction methods for **a** homogeneous phantom and **b** skull phantom. **c**, **d** Temperature errors for the three reconstruction methods compared to “truth”. Note: the three subsampled datasets were sampled with twice the temporal resolution of the fully sampled truth (2.4 vs. 4.8 s) and have been linearly interpolated in time to agree with the fully sampled truth so that the RMSE can be calculated

**Fig. 3:**
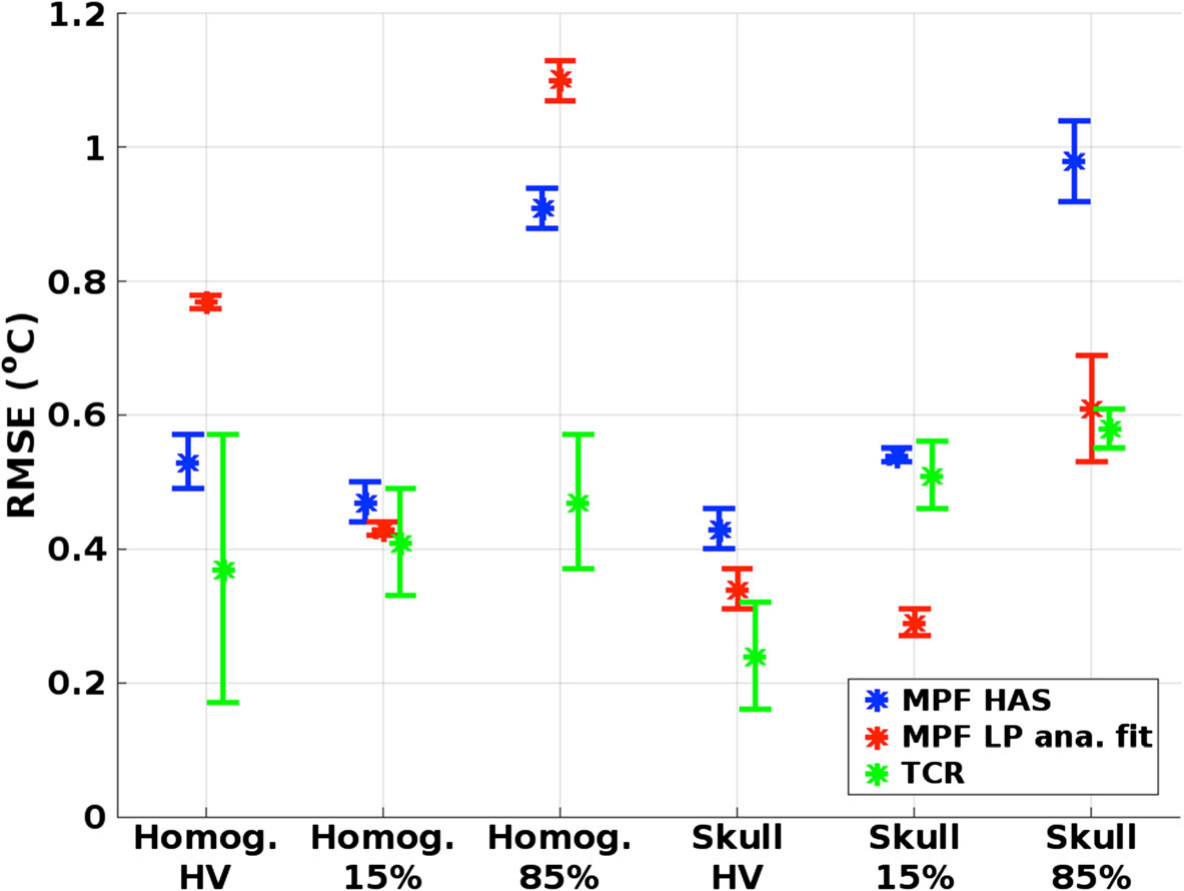
Temperature RMSE. Mean and SD of RMSE of the temperatures as compared to fully sampled “truth”. HV is the RMSE for the hottest voxel. Fifteen percent and 85 % are the RMSE of all voxels that had temperature rises greater than 15 and 85 % of the hottest truth voxel for all time frames. For the homogeneous phantom, this corresponded to temperature rises of 3.3 and 18.7 °C, respectively, and for the skull phantom, this corresponded to temperature rises of 1.8 and 10.1 °C, respectively

**Fig. 4:**
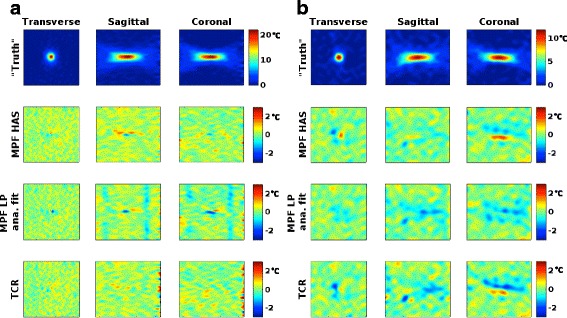
Orthogonal MRTI views of focal spot. Three orthogonal views over the focal spot for the fully sampled “truth” (*top row*), and the error compared to truth for the three reconstruction methods (*bottom three rows*) for **a** the homogeneous phantom and **b** the skull phantom

The mean and SD of the temperature rise of the hottest voxel versus time for the two phantoms can be seen in Fig. 2a, b, respectively. To be able to calculate the RMSE, the subsampled temperatures were linearly interpolated to agree in time with the fully sampled truth as described above, and Fig. 2a, b plots these, lower temporal resolution, interpolated values. Figure 2c, d shows errors for the two phantoms, as compared to the fully sampled truth. The TCR reconstruction can be seen to perform slightly better than the two MPF reconstructions, and this is also seen in Fig. 3, which shows the mean and SD of the RMSE for this hottest voxel for the three reconstruction methods. Figure 3 further shows the RMSE for all voxels with temperature rises greater than 15 and 85 % of the hottest truth voxels in the two phantoms. In general, TCR can be seen to be more accurate than the two MPF methods. Comparing the two MPF methods, MPF HAS is generally more accurate in the homogeneous phantom whereas MPF LP analytical fit is more accurate in the skull phantom. All RMSE for all reconstruction methods were below 1.1 °C. Figure 4 shows the spatial error over the focal spot in one of the three repeated sonications for the three reconstruction methods, at the time of maximum temperature rise.

**Fig. 5:**
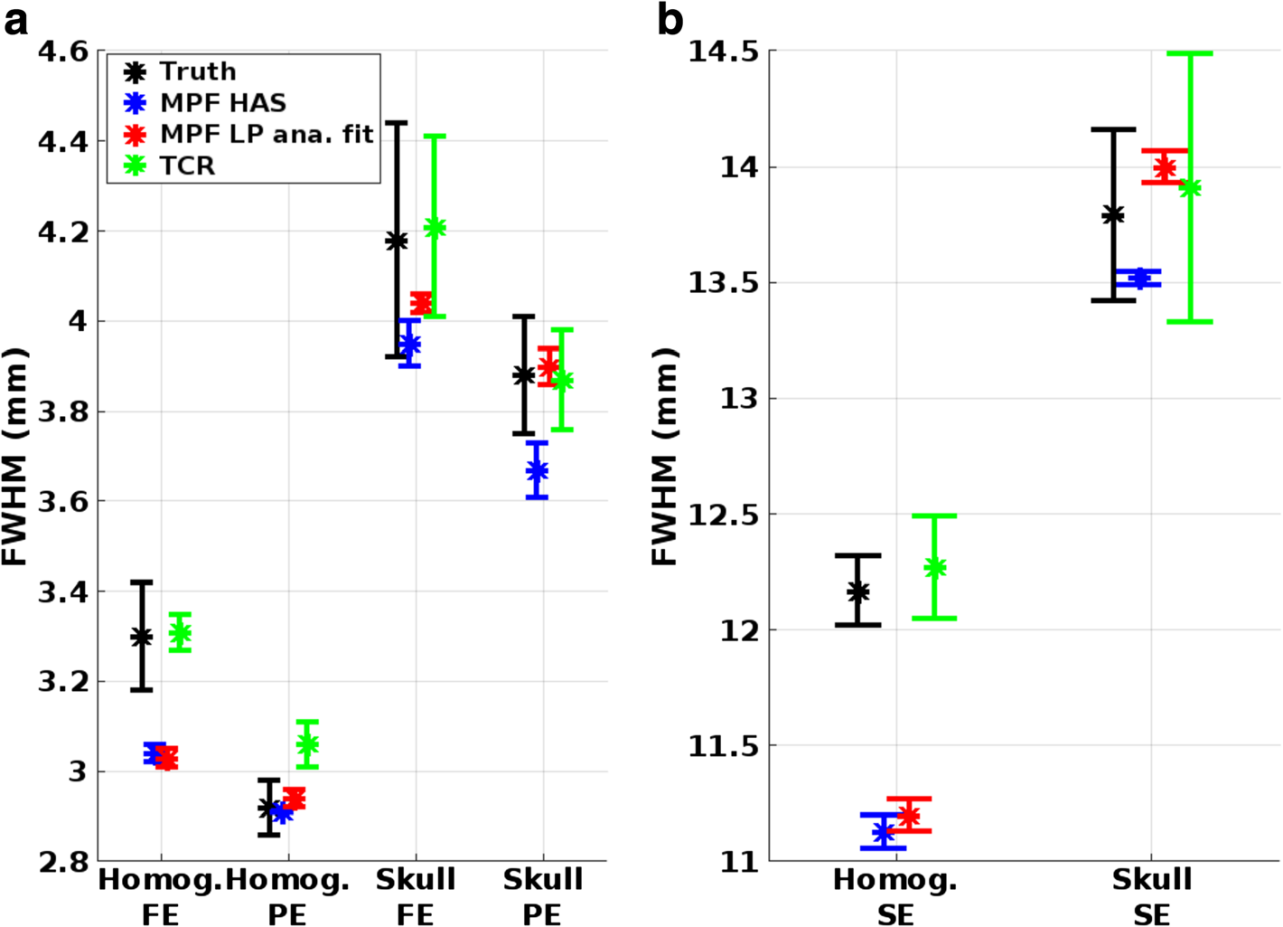
Temperature FWHMs. Mean and SD of FWHM of the temperature profiles in the two phantoms in **a** the frequency encoding (FE) and phase encoding (PE) directions and **b** the slice encoding (SE) direction

Figure 5 shows the FWHM of the temperature profiles in the three orthogonal encoding directions for truth and the three reconstruction methods. For the homogeneous phantom, the largest in-plane (i.e., in the FE-PE-plane) difference between truth and any of the reconstruction methods is below 0.3 mm (largest for MPF LP analytical fit in FE) and the largest through-plane (i.e., in the SE-direction) difference is 1.0 mm (MPF HAS). It can be noted that all these are within the acquired voxel size (i.e., before zero filling, Table 1). For the skull phantom, the widths in all three orthogonal directions also agree well with truth, and the largest differences are 0.2 mm in-plane (MPF HAS in FE) and 0.3 mm through-plane (MPF HAS). It can further be seen that the focal spot is wider in the skull phantom than in the homogeneous phantom, which is to be expected due to phase aberration when focusing through the skull.

**Fig. 6:**
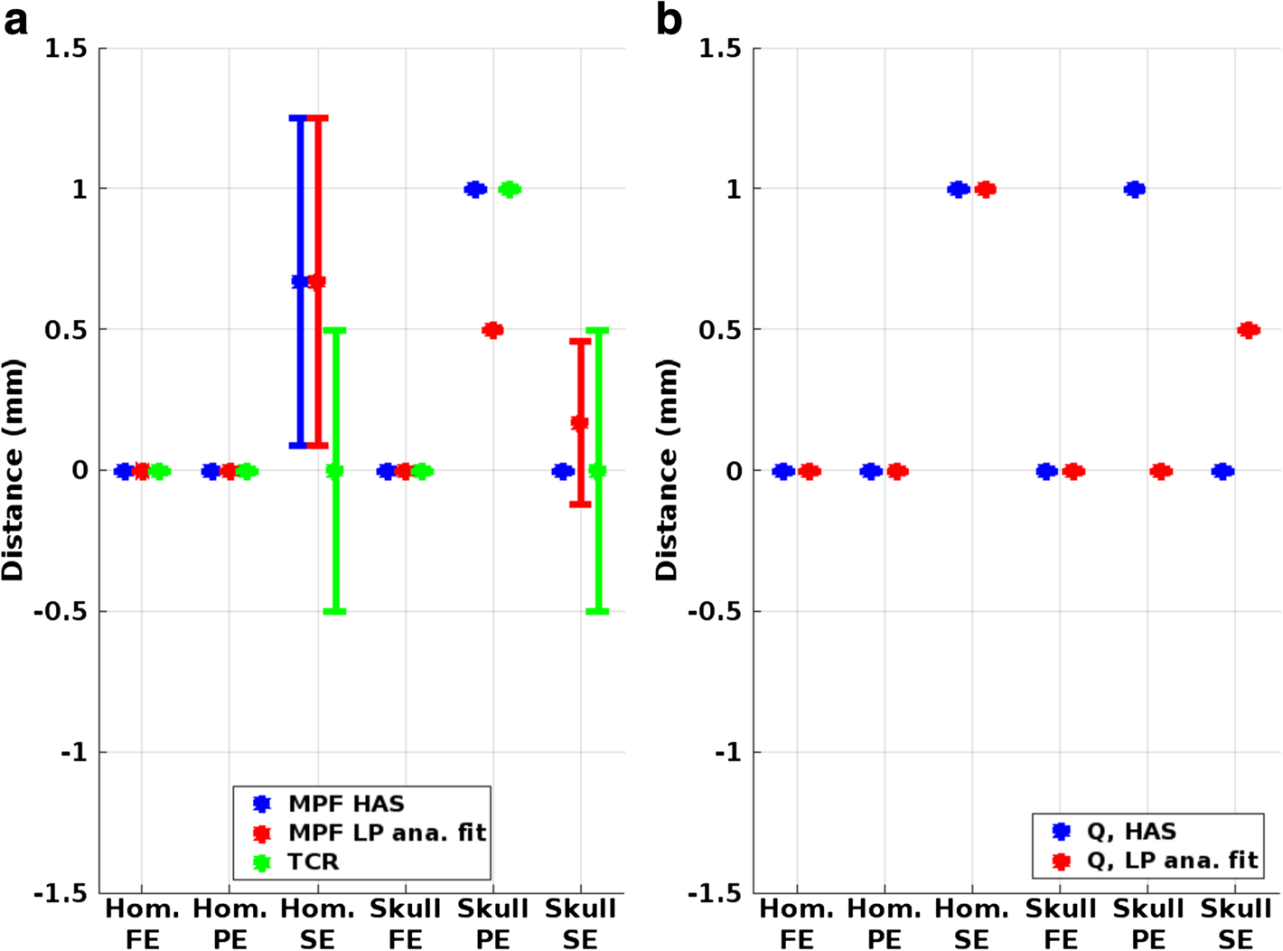
Focal spot position offset. **a** Mean and SD of distance between the hottest “truth” temperature voxel and the hottest temperature voxel in each of the three reconstruction methods in three orthogonal directions (*FE* frequency encoding, *PE* phase encoding, and *SE* slice encoding direction). **b** Distance between hottest “true” temperature voxel and the voxel with maximum *Q*, for *Q* determined using HAS (*blue*) and the analytical solution (*red*)

The distance between the hottest truth voxel and the focal spot center for the MPF and TCR temperature reconstructions is shown in Fig. 6a. Figure 6b shows the distance between the hottest truth temperature voxel and the position of the hottest *Q*-voxel for HAS prediction and LP analytical fit. For most cases, the focal spot positions coincide, and in the cases when the distance is non-zero, it is still within the acquired voxel dimensions.

**Fig. 7:**
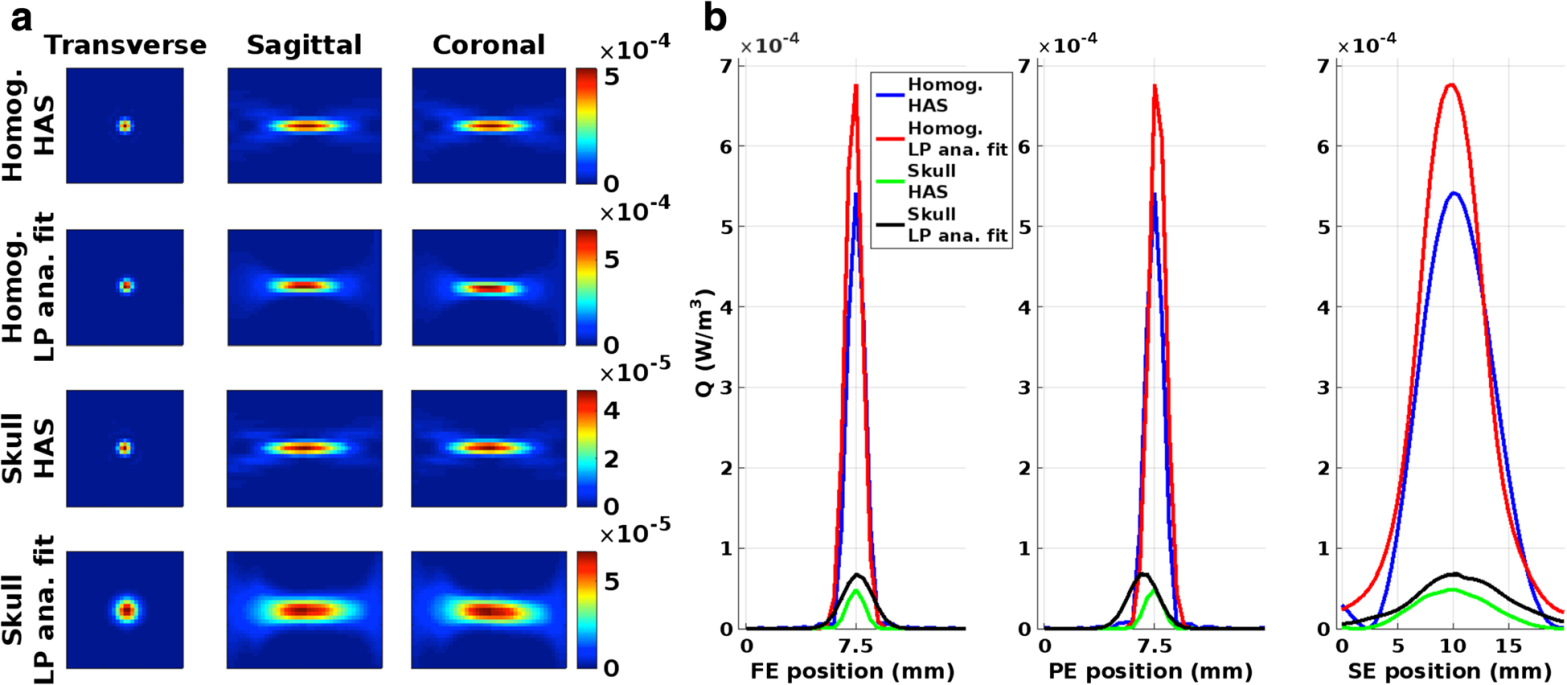
*Q*-patterns. **a** Three orthogonal views of *Q* as derived from HAS and the analytical solution for the homogeneous phantom (*top two rows*) and for the skull phantom (*bottom two rows*), respectively. Note the different color scales. **b** Line plots through the position of the maximum *Q* voxel in all three directions for the four different *Q*s

Figure 7 shows three orthogonal views of the spatial distribution of *Q* for the two methods in determining *Q* (i.e., through HAS simulations and through LP analytical fit) for the two phantoms, and corresponding line plots through the maximum *Q* values. The maximum *Q* values and the FWHM are listed in Table 2. As can be expected, the *Q* in the skull phantom is considerably lower (approximately an order of magnitude) than in the homogeneous phantom since the US is transmitted through the skull, which has higher US absorption than the gelatin. The *Q* prediction from the LP analytical fit is also higher in both phantoms, 25 % higher in the homogeneous phantom and 38 % higher in the skull phantom. In the homogeneous phantom, the widths of the two *Q*-patterns agree to within the measured accuracy, but HAS simulated *Q* is approximately 1 mm longer in the through-plane direction. In the skull phantom, the differences are greater with the HAS-simulated *Q* being approximately 1 mm narrower in-plane and 1.2 mm shorter through-plane.

**Table 2:** Maximum value and FWHM of the Q-patterns for the analytical fit and for the HAS simulation, as obtained in the two phantoms

		Max *Q* (W/m^3^)	FWHM FE (mm)	FWHM PE (mm)	FWHM SE (mm)
Homogeneous phantom	HAS	5.42 × 10^−4^	1.4	1.4	7.7
	LP analytical fit	6.77 × 10^−4^	1.4	1.4	6.7
Skull phantom	HAS	4.87 × 10^−5^	1.4	1.5	8.1
	LP analytical fit	6.74 × 10^−5^	2.4	2.4	9.3

*FE* frequency encoding direction, *PE* phase encoding direction, and *SE* slice encoding direction

For all MPF reconstructions using HAS-simulated *Q,* a tabular value of *k* = 5.49 × 10^−4^ W/(m*°C) was used for thermal conductivity [54], whereas the analytical fit of the low temperature rise heatings for the homogeneous and skull phantom resulted in thermal conductivity values of *k* = 6.50 × 10^−4^ W/(m*°C) and *k* = 7.06 × 10^−4^ W/(m*°C), respectively.

**Fig. 8:**
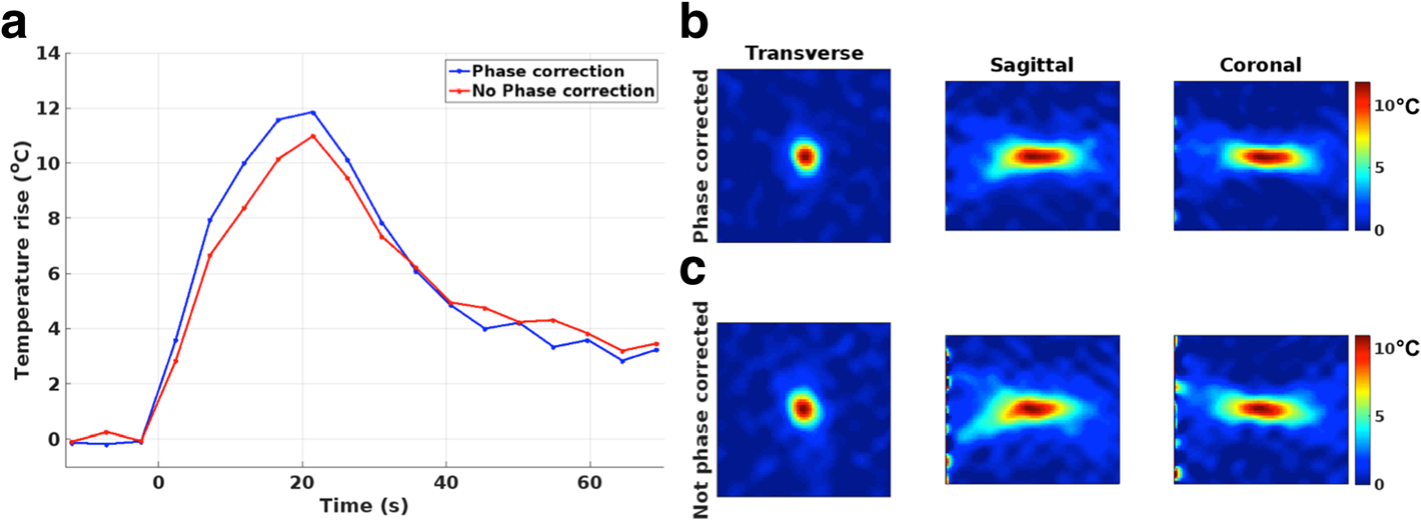
The effects of aberration correction on MRTI. Comparison of sonications performed with and without phase aberration correction. **a** Temperature rise versus time and **b**, **c** three orthogonal views through the focal spot at the time point of maximum heating for phase corrected and no phase correction, respectively. The phase corrected data can be seen to reach a higher maximum temperature, and have a less diffused focal spot

The effect of the phase aberration correction can be seen in Fig. 8, where a comparison of temperature maps from identical sonications monitored with fully sampled acquisitions are shown. The FWHM of the temperature distribution in the non-corrected data was 4.8, 4.2, and 14.0 mm (in FE, PE, and SE directions), which can be compared to the narrower FWHM for the aberration corrected truth seen in Fig. 5 (4.2, 3.9, and 13.8 mm). The maximum temperature rise also increased by 8 %, from 11.0 to 11.9 °C, when the aberration correction was applied.

## Discussion

In this proof-of-concept study, we have investigated the use of US simulations to estimate the power deposition density *Q* for use in the thermal modeling of the MPF method. The temperature maps reconstructed with the HAS-simulated *Q* were, in general, as accurate as those reconstructed with MPF using the empirically determined *Q* from the LP analytical fit, and all temperature RMSE were below 1 °C. Both the position and the shape of the focal spots from the HAS-based temperature maps agreed well with fully sampled truth, and were all within the size of one acquisition voxel (before zero-filling). These results demonstrate the feasibility of using US simulations in conjunction with model-based reconstruction to achieve high spatio-temporal resolution, large-FOV MRTI.

Figures 2, 3, and 4 show that MPF HAS performs as well as or better than MPF LP analytical fit for the case of the homogeneous phantom. This mainly seems to be due to a smaller overestimation of the temperatures (Fig. 2c), as Figs. 5 and 6a show very similar widths and positions of the focal spots compared to truth. There is also a relatively small difference in the widths of the predicted *Q*-patterns (Table 2), and their locations coincide with within the zero-filled resolution of 0.5 mm. There is, however, a relatively large difference of 25 % in the maximum *Q* value (Table 2), but the higher *Q* estimated for the LP analytical fit solution is counteracted by an 18 % higher estimated *k* than the tabular value used in MPF HAS (6.50 compared to 5.49 W/(m*°C)). Comparing the offsets in temperature and in position of *Q* (Fig. 6a compared to Fig. 6b), it can further be observed that the temperatures more accurately predict the focal spot location since they are based on a combination of the model (which depends on *Q*) and the acquired MR data.

For the more challenging case of the skull phantom, MPF HAS slightly underestimates the temperatures during the heating whereas MPF LP analytical fit overestimates the temperatures (Fig. 2d), and in general, MPF LP analytical fit is more accurate (Fig. 3). This seems to be mainly due to a narrower focal spot in all three directions for MPF HAS compared to MPF LP analytical fit and truth (Fig. 5) and not due to a mismatch in the actual position of the focal spot, where all three reconstruction methods are within 0.5 mm (Fig. 6a). The underestimation in the width of the MPF HAS focal spot can be attributed to the narrower *Q* from the HAS simulations: approximately 1 mm in all three directions as compared to the LP analytical fit solution (Table 2). Just as for the homogeneous phantom, MPF LP analytical fit estimates higher *Q* than HAS (38 %) and the LP analytical fit solution correspondingly also results in a compensating higher *k*, in this case, approximately 29 % higher.

The current study intended to compare MPF HAS to MPF LP analytical fit and since the analytical fit method assumes a Gaussian-shaped focal spot, the comparison and error calculations were focused on the focal spot (i.e., not including off-focal regions). Although no truth for the US near- and far-field (e.g. along the “brain” surface in the skull phantom) was available in this study, and hence, RMSEs could not be calculated, it can be noted that outside of the focal spot region where *Q* was set to zero (and hence, the thermal modeling contribution will be zero), the MPF method will reconstruct temperature maps using a “sliding window” or “view sharing” of the subsampled k-space. This ensures that no off-focal heating will go undetected, and as long as any off-focal heating is relatively slow (i.e., low dT/dt), it can be accurately monitored with the sliding window reconstruction. For the sliding window reconstruction to work properly, the perfusion and thermal conductivity (i.e., *W* and *k*) are also set to zero for the off-focal regions, so that no heat dissipation is modeled. It can further be noted that HAS predicts power deposition for off-focal areas which could be used for MPF reconstructions of, e.g., the US near-field. The accuracy and precision of this will be investigated in future studies.

The accuracy of the position of the *Q*-patterns determined with HAS makes it a promising approach for pre-treatment localization of the focal spot. In many current applications, such as transcranial treatments, initial low-power sonications monitored with MRTI are performed to evaluate the exact position of the focal spot [59, 60]. With accurate US simulations, this could possibly be performed without the potentially tissue-damaging pre-treatment sonications. The achieved accuracy of approximately 0.5–1.0 mm in Fig. 6 is on the order of the current MRTI voxel size and can be deemed adequate. Alternative ways to localize the focal spot are through MR acoustic radiation force imaging (AFRI) or possibly using so called MR tracker coils. In MR-ARFI, low-energy US sonications are used to encode tissue displacement into the MR phase image, which can then be used to localize the focal spot [61–64]. Just as for MRTI, the sonication needed for ARFI can potentially be tissue damaging [65, 66]. For situations where the ultrasound transducer can be moved relative to the patient, MR tracker coils on the transducer can be used to triangulate the intended focal spot position without the need for an actual sonication [67, 68]. However, unlike the alternative methods (i.e., US simulation, MRTI, and MR-ARFI), tracker coils will not work when focusing through an aberrating medium such as the skull bone. US simulation-based focal spot localization hence has the potential to both avoid the pre-treatment sonication and work in, e.g., transcranial applications.

The fact that the magnitude of the *Q*-patterns can differ between 25 and 38 % for the HAS and the LP analytical fit method, as seen in Fig. 7, and that accurate temperature maps can still be reconstructed highlights the robustness of the MPF method. As was previously shown, the accuracy depends on both the amount of k-space subsampling (*R*) and the rate of temperature increase (*dT/dt*) [47]. A relatively high subsampling factor of *R* = 7 was used in this study (the previous study showed that subsampling factors up to *R* = 12 produced accurate temperature measurements ex vivo), and a lower *R* could be used to achieve more accurate temperature measurements.

As expected, the plastic skull attenuated the US considerably, and both the HAS-simulated and analytically derived *Q* were at least an order of magnitude lower in the skull phantom than in the homogeneous phantom. A more than three times increase in US power (125 versus 40 W used in the skull and homogeneous phantoms, respectively) resulted in only about half the temperature rise (max ΔT of 11.9 versus 22.0 °C in the skull and homogeneous US phantoms, respectively). This difference of approximately 6× (3× the power resulting in 0.5× the temperature rise) agrees well with previous hydrophone studies showing an approximate 85 % drop in US intensity when focusing through the skull, compared to focusing in water [69].

The fact that HAS simulations did not include US scattering may be part of the reason for the narrower simulated *Q* compared to the *Q* from the LP analytical fit solution. Other factors affecting the larger discrepancies seen in the skull phantom can include uncertainties in the tabular acoustic values used for the skull, where, for example, an underestimation of the speed of sound would result in an underestimated wavelength and hence beam width. The accuracy of the skull segmentation, which for in vivo applications routinely is performed based on high-resolution computer tomography (CT) images with sub-millimeter resolution [60], can also be assumed to play a role.

This study was performed in relatively simple phantoms, and further in vivo validation studies are needed. Possible challenges related to application in vivo include inhomogeneous tissues and more complex anatomies. Both HAS and MPF can readily handle inhomogeneous tissues, but the effect on MRTI accuracy will have to be investigated. For in vivo applications, blood perfusion will also have to be taken into account, and even though perfusion is relatively low in the brain and (resting) muscle tissue, it can act as a significant heat sink in tumor tissue, near blood vessels, and in organs such as the kidney. For transcranial applications, the challenge of more complex composition of the skull bone will also have to be accounted for (the plastic skull used in this study had varying thickness, but real bone will also have varying density between cortical and trabecular bone). Current clinical transcranial treatments make use of high-resolution CT scans [60] for phase aberration correction, but recent studies have also shown that MR imaging from both ultrashort echo time (UTE) and T1-weighted pulse sequences may achieve similar accuracy [70, 71].

Despite the challenges that arise when going to in vivo applications, recent studies using HAS to simulate the US field from a clinical tcMRgFUS system inside the human skull, in addition to using these simulations for thermal modeling utilizing PBTE, show promising results [72, 73]. This, together with results presented here and previously [47] showing that the MPF method is relatively robust to errors in *Q* and *k* makes it seem possible that accurate in vivo temperature maps can be achieved.

## Conclusions

This proof-of-concept study has shown that it is possible to utilize US simulations from HAS to accurately estimate the US power density deposition *Q*, and further use the estimated *Q* to reconstruct high spatio-temporal resolution temperature maps covering large FOVs. The reconstructed temperature maps were accurate for both a homogeneous phantom and when focusing the US through a plastic skull. Future studies will aim at improving the HAS simulations through inclusion of US scattering and in vivo validation of the described methods.

## Abbreviations

3D: Three dimensional
bSSFP: Balanced steady state free precession
BW: Bandwidth
EPI: Echo planar imaging
ES: Echo spacing
ETL: Echo train length
FA: Flip angle
FDTD: Finite-difference time-domain
FE: Frequency encoding
FOV: Field of view
FUS: Focused ultrasound
FWHM: Full width at half maximum
GRE: Gradient recalled echo
HAS: Hybrid angular spectrum
HV: Hottest voxel
LP: Low power
MPF: Model predictive filtering
MR: Magnetic resonance
MRI: Magnetic resonance imaging
MRTI: Magnetic resonance temperature imaging
PBTE: Pennes bioheat transfer equation
PE: Phase encoding
PRFS: Proton-resonance frequency shift
R: Reduction factor
RF: Radiofrequency
RMSE: Root-mean-square error
SD: Standard deviation
SE: Slice encoding
SNR: Signal-to-noise ratio
t_acq_: Acquisition time
TE: Echo time
TR: Repetition time
US: Ultrasound
UTE: Ultrashort echo time
